# Functional Electrical Stimulation in Conjunction With Proprioceptive Neuromuscular Facilitation (PNF) Technique to Improve Upper Limb Function in Traumatic Brachial Plexus Injury: A Case Report

**DOI:** 10.7759/cureus.46386

**Published:** 2023-10-02

**Authors:** Nishigandha P Deodhe, Pooja Dhage, Pallavi Harjpal

**Affiliations:** 1 Neurophysiotherapy, Ravi Nair Physiotherapy College, Datta Meghe Institute of Higher Education and Research, Wardha, IND; 2 Musculoskeletal Physiotherapy, Ravi Nair Physiotherapy College, Datta Meghe Institute of Higher Education and Research, Wardha, IND; 3 Physiotherapy, Ravi Nair Physiotherapy College, Datta Meghe Institute of Higher Education and Research, Wardha, IND

**Keywords:** tbpi, upper extremity functional index, brachial assessment tool, upper extremity, functional electrical stimulation, proprioceptive neuromuscular facilitation

## Abstract

Traumatic brachial plexus injuries (TBPIs) in the adult population are primarily a result of road traffic accidents or falls on a shoulder, which mainly affects the young population. Adult TBPI is a serious incapacitating injury that affects young adults. It decreases the function of upper extremity muscles, which affects social participation and quality of life. Physiotherapy intervention demonstrates its effectiveness in enhancing and maintaining the function of the upper extremity, eventually decreasing the participation restriction and improving quality of life. The proprioceptive neuromuscular facilitation (PNF) technique has been selected as a useful therapeutic option to enhance upper limb function after TBPI. The preceding case report proved the effectiveness of six weeks of functional electrical stimulation in addition to the PNF technique in improving upper limb function after TBPI.

## Introduction

Plexus brachial is a web of nerves that provide movements and a sense of touch to the entire upper extremity (UE). Traumatic brachial plexus injuries (TBPIs) are caused mainly as a result of trauma by motorcycle accidents, sports activities, and falls on the shoulder [1,2]. The prevalence of brachial plexus injury (BPI) is 5.74%. It is more common in adult males aged between 24 and 64 years, that is, 90.5%. Seventy-two percent of BPI cases mainly occur as a result of motor vehicle accidents [3]. Adult TBPIs are devastating injuries. They result in physical disability, psychological suffering, and socioeconomic deprivation [4]. Closed BPIs are generally related to a traction mechanism in which the arm and shoulder are forcibly distracted away from the neck and trunk.

In contrast, open BPIs mainly occur as an effect of a gunshot injury, stab injury, and sometimes an open fracture associated with the shoulder girdle. Patients typically have a loss of sensation and muscle power and may have episodes of disabling neuropathic pain [5]. TBPI leads to loss of UE function, leading to disability, limitations, and participation restrictions [6]. The alteration in the kinematics of the UE leads to the affection of coordination of the injured hand [7]. The TBPIs are destructive; they not only result in sensory and motor dysfunction but also have a notable effect on quality of life. TBPI decreases the patient’s capacity to fulfill occupation-associated work and everyday activities. It ultimately affects their behavioral and psychological function. It potentially affects the quality of life and leads to socioeconomic hardships [7,8].

Rehabilitation serves an essential role in the improvement of function post-trauma. The timely intervention is guaranteed to minimize secondary complications [1,2]. Physiotherapy interventions after TBPI are identified as essential interventions that need to be initiated timely and demand extended periods of treatment [9,10]. Physiotherapy intervention mainly focuses on impairments on a structural and functional level, including components of activity limitations and participation restrictions in their environmental and personal conditions [11]. Various therapeutic interventions have been proven effective in restoring the upper limb function.

Based on context, the proprioceptive neuromuscular facilitation (PNF) concept becomes evident in restorative alternatives. It has shown its effectiveness in improving the function of UE in patients with TBPI. This therapeutic approach can be applied to different types of neurological and musculoskeletal conditions [6]. It is the basis of the neurophysiologic approach to motor control and learning [10]. It utilizes the body’s proprioceptive system to facilitate as well as to inhibit muscle contraction. The definition of PNF encompasses the term proprioceptive (which has to do with sensory receptors that provide information concerning movement and position of the body), neuromuscular (including nerves and muscle), and facilitation (making it easier) [12].

Functional electrical stimulation (FES) was effectively proven as a physical intervention for motor recovery. It is the electrical stimulation of motor neurons such that muscle groups are stimulated to contract and augment a moment around the joint. FES proved its effectiveness in increasing the perfusion to the same side as a lesion of the sensory and motor cortex and also leads to cortex excitability [11]. These findings indicate a more significant potential for voluntary FES to induce muscle contraction through neuroplasticity. FES has been investigated as a treatment intervention focusing explicitly on nerve regeneration following TBPI [13]. It uses low-energy electrical pulses to initiate muscle contraction in disabled muscles. It is a short-term therapy. It is used to generate muscle contractions in paralyzed muscles to produce voluntary movements of the upper limb [14]. The present case study aims to elucidate the effect of PNF in conjunction with FES to enhance the function of the upper limb post-TBPI.

## Case presentation

Patient information

A 38-year-old male with a height of 167.64 cm and weight of 51 kg suffered a road traffic accident in August. He fell on his left shoulder. He experienced a superficial head injury, upper back injury, and bruising over the lower limb. He was brought to the hospital on the same day, and his CT scan and X-ray showed a left spine scapula fracture and clavicle fracture with a brachial plexus injury. The fracture was managed with plating in November. After surgery, he was having difficulty in moving his left upper limb. He had pain and a tingling sensation over his left shoulder.

Clinical findings

A patient visited the physiotherapy department after two months as he could not move his left upper limb. Written consent was taken from the patient before the assessment. The patient was cooperative, conscious, and oriented throughout the evaluation session. Superficial sensation, muscle tone, and reflexes were intact in the bilateral upper limb. Muscle power and girth were reduced in the left UE. Manual muscle testing and passive range of motion were assessed (Tables 1-2). On the left side, muscle atrophy was present, and muscle circumference was reduced by 5-6 cm. The patient experienced pain and a tingling sensation over the left shoulder.

**Table 1:** Manual muscle testing.

Muscles	Right	Left
Lower and middle fibers of trapezius	Grade 5	Grade 2
Upper fibers of trapezius	Grade 5	Grade 2
Serratus anterior, rhomboidus, and levator scapulae	Grade 5	Grade 3
Deltoid	Grade 5	Grade 2
Teres minor	Grade 5	Grade 2
Teres major, infraspinatus, subscapularis, supraspinatus	Grade 5	Grade 2
Elbow flexors	Grade 5	Grade 2
Triceps brachii	Grade 4	Grade 2
Wrist flexors	Grade 4	Grade 1
Wrist extensors	Grade 5	Grade 1

**Table 2:** Passive range of motion.

Movement	Left	Right
Shoulder flexion	0-90°	0-180°
Shoulder abduction	0-95°	0-180°
Elbow flexion	20°-125°	0-135°
Elbow extension	125°-20°	135°-0
Wrist flexion	0-80°	0-90°
Wrist extension	0-10°	0-70°
Radial deviation	0-15°	0-20°
Ulnar deviation	0-25°	0-30°

Investigations

**Figure 1:**
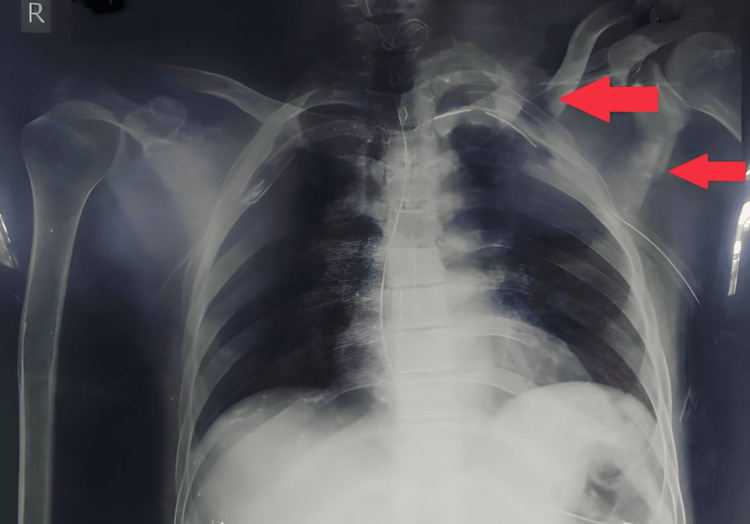
Preoperative X-rays of fracture of the middle third of the clavicle and scapula.

**Figure 2:**
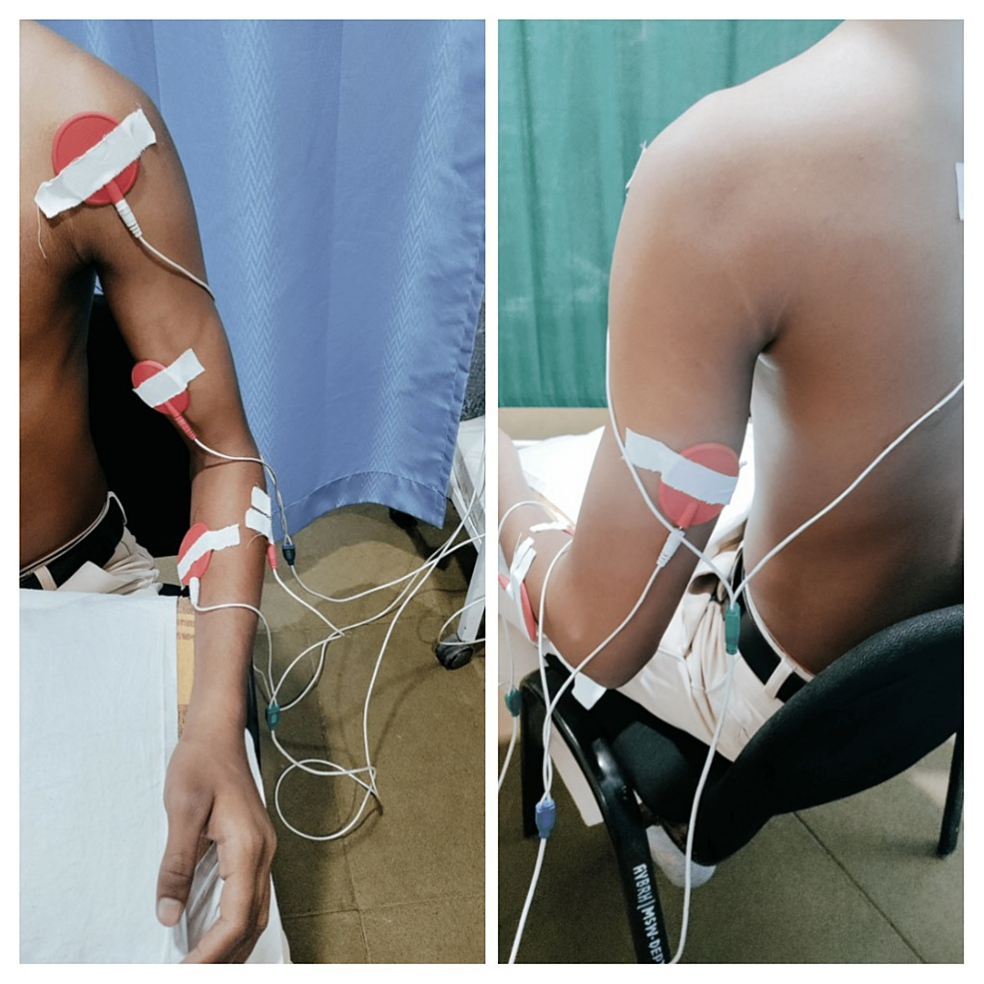
Placement of FES electrodes. FES, functional electrical stimulation

Preoperative X-rays reveal the fractures of the middle third of the clavicle (left) and scapula (left) (Figure 1) and postoperative X-rays of the middle third of the clavicle with open reduction and internal fixation (ORIF) and scapula with scapula platting (Figure 2).

Therapeutic intervention

The patient was educated about his condition and informed about the findings, physical assessment results, and recommended course of therapy. The patient was given FES, a rhythmic initiation technique, and a combination of isotonic PNF to the left upper extremity to enhance the strength of the muscle and joint mobility. The rhythmic initiation and a combination of isotonic techniques were exercised in all patterns. Rhythmic initiation teaches movement, helps the patient to calm and relax, improves coordination, and increases mobility. The combination of isotonic enhances strength and coordination. The exercises are performed in two sets with five to eight exercise repetitions with 1-1-minute intervals between each set of the same exercise and 2 minutes of rest between one type of exercise to another. The interscapular muscles, triceps brachii, anterior deltoid, biceps brachii, wrist extensors, and flexors are selected for FES stimulation in the UE (Figures 1-2). The stimulation parameters of FES include pulse amplitude (10-100 mA), pulse frequency (0-60 Hz), pulse width (0-360 microseconds), total output time (0-6 seconds), and time from zero to maximum amplitude (0-2 seconds). The intervention was given for a five-day session with 20 minutes of stimulation for six weeks.

Outcome measures

Brachial assessment tool (BrAT) and upper extremity functional index (UEFI) were used to evaluate UE function before and after intervention. BrAT is a self-reported outcome measure designed to evaluate the activity after TBPI. The test-retest reliability was excellent (0.90-0.97). It is the 31-item outcome measure created to draw attention to the issues of the affected extremity. It includes four responses to each item. It is used as three separate subscales, which include (1) eight items of dressing and grooming, (2) 17 items of upper limb including arm and hand, and (3) six items of no hand; or alternatively. These 31-item scores are calculated to generate the summed score. The BrAT item responses are scored as 0 (cannot do now), 1 (very hard and slow), 2 (a little hard to do now), and 3 (easy to do now).

UEFI is an outcome measure used to quantify activity limitations due to UE disorders. The 20 items were scored from 0 (extremely difficult or unable to perform activity) to 4 (no difficulty), and the total score ranged from 0 (worst function) to 80 (best function). It shows the best test-retest reliability. The findings indicate significant improvement in the outcome measure after six weeks of intervention (Table 3).

**Table 3:** Pre- and postintervention scores of outcome measures. BrAT, brachial assessment tool; UEFI, upper extremity functional index

Outcome measures	Preintervention	Postintervention
BrAT	45	55
UEFI	30	45

## Discussion

TBPI causes severe functional limitations. It impairs the daily activities performed by the UE. TBPI is caused by forceful events that lead to the loss of function of UE [1,5]. Physiotherapeutic intervention has been proven effective in increasing the UE function. This case study intends to show the effectiveness of FES and the PNF technique in improving upper limb function. BrAT and UEFI were used as outcome measures to assess the UE function. Studies showed the effectiveness of PNF in recovery of the upper arm function after crushing and avulsion injury [6,9,10,15]. This study showed the effect of PNF techniques that aim to regain functionality through a neurofacilitation approach by remodeling the cortex through motor control and motor learning principles [16]. Application of these techniques helps to commence and produce learning of new movements. Rhythmic initiation and a combination of isotonic is the suggested approach technique to learn new motion. PNF position enhances the joint range of motion and muscle power by changing the muscle discharge order [6,12,15].

FES has promising results on the primary outcome of activities of daily living (ADL) associated with the UE function [17]. Electrical stimulation enhances axonal regeneration and functional recovery following TBPI [11]. Electrical stimulation and exercise enhance axonal regeneration, increase neural activity, and promote nerve regeneration [18]. Peripheral axotomy elicits growth-associated gene programs in sensory and motor neurons that can support the reinnervation of peripheral targets, given sufficient levels of debris clearance and proximity to the nerve target [19]. FES has shown its efficacy in promoting regeneration and reducing the denervating skeletal muscles' atrophy [20]. FES is used to evoke electrical stimulation in a specific sequence and magnitude, which is used to create the muscle activity required to perform a functional task [21]. FES treatment early in the stroke can decrease the chances of shoulder subluxation [12,21].

## Conclusions

It becomes difficult for the patients to carry out everyday activities with decreased UE function. PNF and FES have shown convincing recovery in the UE function after six weeks of intervention. Thus, PNF in combination with FES can be considered as a clinically effective intervention to improve functional mobility following TBPI.
